# Evaluation of tensile properties of spherical shaped SiC inclusions inside recycled HDPE matrix using FEM based representative volume element approach

**DOI:** 10.1016/j.heliyon.2023.e14034

**Published:** 2023-02-26

**Authors:** Santosh Kumar Sahu, P.S. Rama Sreekanth

**Affiliations:** School of Mechanical Engineering, VIT-AP University, Inavolu, Besides AP Secretariat, Amaravati, Andhra Pradesh, 522237, India

**Keywords:** Polymer matrix composite (PMC), Representative volume element (RVE), Micromechanics, Tensile modulus

## Abstract

In the current study, a FEM-based representative volume element (RVE) technique is used to evaluate the elastic modulus of recycled high-density polyethylene (rHDPE) filled spherical-shaped shaped silicon carbide (SiC). In the ANSYS 2019, the material designer (MD) module is used to generate a 3D RVE of 500 × 500 × 500 μm cuboid, with randomly dispersed spherical SiC particles (i.e., 10, 15, 20, and 30% volume fractions) inside rHDPE. The Young's modulus values extracted from the RVE technique at various volume % are substantially nearer to experimental data than other micromechanical models. The tensile performance of the composite is simulated, and it was noted that the maximum equivalent stress of 4.1133 MPa for rHDPE/30% SiC composite, which is decreased to 13.8, 7.8 and 6.8% for rHDPE/10% SiC, rHDPE/15% SiC and rHDPE/20% SiC composite respectively. The results are astounding for immediate application in the relevant field of interest.

## 1 Introduction

In the modern world, excessive single-use plastic use contributes to global warming since more fossil fuels are required for plastic production, which will accelerate the effects of climate change. Worldwide, over 8.3 billion tonnes of plastic are manufactured, yet only 9% are recycled; instead, 60% end up in landfills or as trash. Scientists and environmentalists are expressing concern about the rising use of plastics, which endangers habitats and aquatic life. However, the world has gradually begun to recognize this as a severe threat to humanity over the years. It has initiated strict regulations for lowering the use of plastics and their appropriate upcycling [1]. Waste management techniques such as the four R's (reduce, reuse, recycle, and recover) concentrate on environmentally friendly ways to save resources and curtail energy usage, creating a circular economy. Although there are many types of recyclable plastic available for engineering use, however recyclable high-density polyethylene (rHDPE) is the best choice owing to its low cost [[2], [3], [4]], ease of processing [[5], [6], [7]], superior mechanical [8,9], tribological [10,11], and thermal properties [12]. Compared to virgin rHDPE, addition of reinforcement, the above properties may enhance enormously [13]. The following section briefly discusses the available literature in the relevant area concerned.

Alghamdi [14] reported the influence of environmental aging on the morphological and mechanical behavior of fly ash filled in high-density polyethylene composites (i.e FA/HDPE) (FA: 5, 10, and 15 wt %) and compared the results with virgin HDPE samples. The mechanical properties of recycled HDPE (rHDPE) reinforced with bamboo fiber were analyzed by Widiastuti et al. [15]. It was observed that the tensile strength of the rHDPE material with 0 wt % fiber loading is 8.3 N/mm^2^, which is higher than that of 30 wt % bamboo fiber. This was reasoned to the absence of adhesive substances and the randomness of bamboo fiber directions. In their research, al-Talib et al. [16] adopted rice husk reinforcement in rHDPE and accessed the mechanical properties of recycled high-density polyethylene composite (rHDPE/rPET). This study noted an increase of 4.95 and 162.65% in tensile strength and elastic modulus compared to the pure recycled thermoplastic material. Satya et al. [17] investigated rHDPE reinforced with graphene and MWCNT nanoparticles (0.1, 0.3, and 0.5% by wt. %). It was observed that adding fillers into recycled HDPE substantially improved the mechanical properties. Toroslu [18] reported on the tensile properties of rHDPE with the inclusion of Al_2_O_3_ (1–7%). The results showed that adding 7% Al2O3 inclusion improved the tensile modulus by 3.74% compared to virgin rHDPE. Diouf et al. [19] demonstrated the use of ronier palm leaf fiber (RLF) (0–40 wt %) in enhancing the tensile properties of rHDPE. It was observed that the composite filled with 40 wt % of RLF, there is an enhancement of 31% in Young's modulus compared to pure rHDPE. Tazi et al. [20] investigated the mechanical properties of HDPE filled with sawdust particles from 0 to 60 wt %. Results showed that the mechanical performance of the composite improvised with the addition of sawdust particles.

Based on the literature study, the works were dedicated towards rHDPE reinforced with fly ash, bamboo fiber, graphene, CNT fillers, etc. However, research on rHDPE filled with spherical-shaped SiC as inclusions is absent in the literature. The authors are keen to investigate the effect of spherical-shaped silicon carbide (SiC) inclusions on the tensile properties of recycled high-density polyethylene (rHDPE) at various volume fractions of SiC. The choice of spherical shaped SiC material owing to superior tensile properties [21], hardness [22,23], and also re-cyclable [24]. The current study's novelty is that conventional research works only focus on experimental aspects for assessing the tensile property. However, the finite element method-based representative volume element (RVE) would help in accurately predicting the tensile property of randomly generated spherical-shaped inclusions [25] in rHDPE, which will save time, conserve energy, and reduces the wastage of material.

## 2 Materials and methods

### 2.1 Materials

**Fig. 1:**
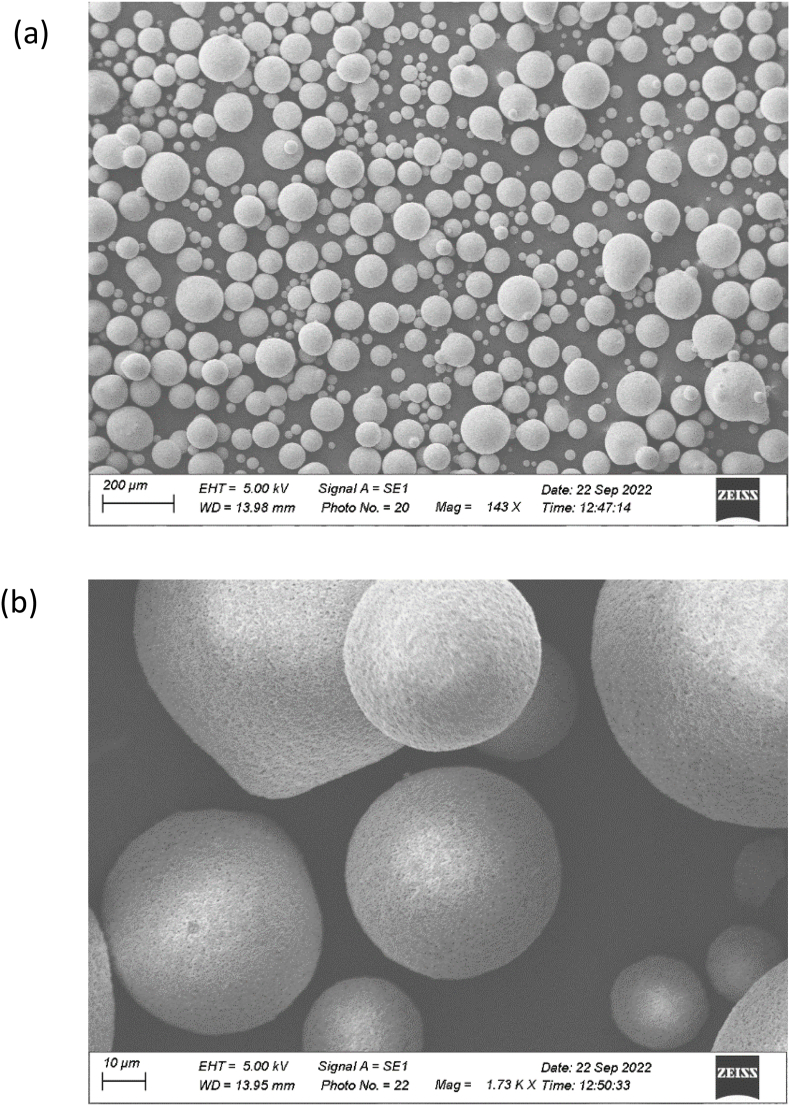
TEM imaging of spherical shaped silicon carbide a) lower resolution; b) higher resolution.

Recycled High-Density Polyethylene (rHDPE) purchased from a local recycler in Vijayawada, India. The spherical-shaped silicon carbide (SiC) material was chosen as filler/inclusion, supplied by Nano Research elements, India. Table 1 shows the detailed specifications of rHDPE and SiC. The morphology of the SiC was a spherical-shaped structure confirmed by Transmission electron microscopy (TEM) imaging, as shown in Fig. 1a and b.

**Table 1:** Materials specification.

Materials	Specification
rHDPE	Pallet form of size 5–8 mm; Melt flow index (MFI) of 20 g/10 min; Melting point of 125–135 °C, Density is 0.956 g/cm^3^; Poison's ratio is 0.41, Young's modulus is 1080 MPa.
Spherical SiC	Particle size (average) < 50 μm, Purity >99.9%, Density 3.1 g/cm^3^; Poisson's ratio is 0.14 and Young's modulus 4000 MPa.

### 2.2 Fabrication of composite

**Fig. 2:**
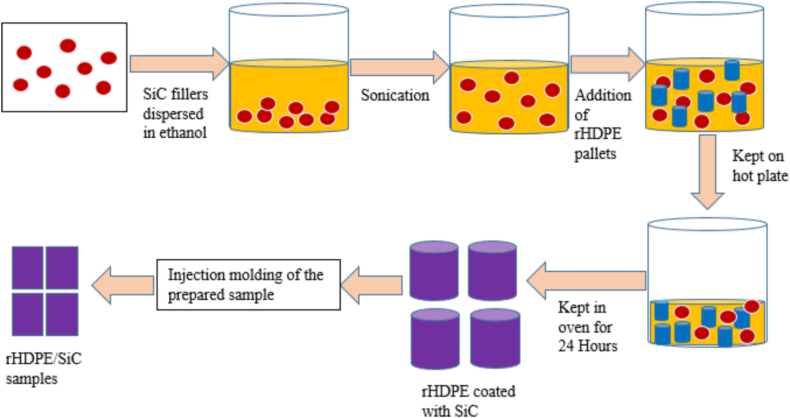
Fabrication route followed.

The composites were fabricated through recycled High-Density Polyethylene (rHDPE) reinforced with 10, 15, 20, and 30 vol % of spherical-shaped silicon carbide (SiC) microparticles. Fig. 2 shows the fabrication route followed for rHDPE/SiC composite fabrication. The foremost step is the chemical modification of the required volume % of SiC as detailed in literature Monton et al. [26], then pouring the required volume fraction chemical modified SiC micro-particle in a beaker having ethanol base (1:0.5 ratio) and stirring well by keeping over a sonicator until a uniform dispersion solution is achieved. The rHPDE pallets are poured into the above solution and stirred well by keeping them on a hot plate. The process is continued until all the ethanol base is evaporated. Consequently, after evaporation of all the ethanol, rHDPE pallets will be coated with the required volume % of SiC microparticles.

To ensure no tress of moisture, the samples are stored in an oven for 24 h at 100 °C. The samples are then poured through a hopper on an injection molding machine to obtain a sample in a rectangular sheet. The samples were then cut as per ASTM standard D1708. A similar procedure was adopted for all the samples, and the samples were named rHDPE/10% SiC, rHDPE/15% SiC, rHDPE/20% SiC, and rHDPE/30% SiC.

### 2.3 Tensile test

Tensile testing was performed using Instron 8801 UTM machine to evaluate Young's modulus for all the fabricated samples according to ASTM D1708. The UTM has a load range of ±100 kN and is tested under normal atmospheric conditions. For each sample, five samples were tested, and the average value is noted.

### 2.4 Micromechanical models

The micromechanical models, such as Rule of mixture (ROM), Reuss (REU), and Voight (VOI) were used to determine Young's modulus of rHDPE/SiC composites (i.e 10, 15, 20, and 30 vol % of SiC fillers). The following equations (1), (2), (3)) were useful [27]- Rule of mixture (ROM)

(1) $$E_{c}=\frac{E_{m}E_{f}}{E_{m}V_{m}+E_{f}V_{f}}$$

Reuss (REU)

(2) $$\frac{1}{E_{c}}=\frac{V_{f}}{E_{f}}+\frac{\left(1-V_{f}\right)}{E_{m}}$$

Voight (VOI)

(3) $$E_{c}=V_{f}E_{f}+\left(1-V_{f}\right)E_{m}$$

The symbols above refer to Daramola et al. [27].

### 2.5 RVE, FEM, and boundary conditions details

**Fig. 3:**
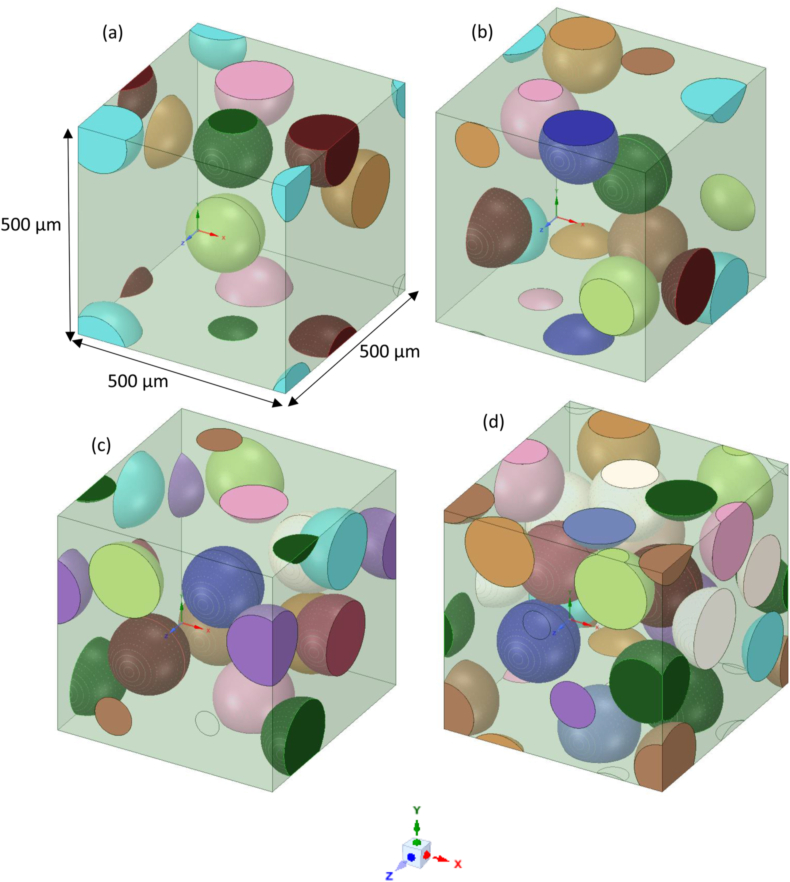
Generated 3D RVE of a) rHDPE/10%SiC; b) rHDPE/15%SiC; c) rHDPE/20%SiC; d) rHDPE/30% SiC.

The ANSYS 2019 FEM software is used to create a representative volume element (RVE) of composites. In the workbench GUI, the material designer module section was chosen to generate 3D microstructure RVE of rHDPE/SiC composite. Fig. 3a–d shows the generated 3D representative microstructure of the composites, i.e., rHDPE/10%SiC, rHDPE/15%SiC, rHDPE/20%SiC and rHDPE/30% SiC respectively. The RVE is made through a 500 × 500 × 500 μm cuboid structure to predict Young's modulus for all the composites. The FEM-based RVE uses equations (4), (5), (6), (7), (8), (9)) for the computation [28]-

(4) $$\bar{\sigma_{ij}}=\frac{1}{V}\int_{V}\sigma_{ij}dV$$

(5) $$\bar{\varepsilon_{ij}}=\frac{1}{V}\int_{V}\varepsilon_{ij}dV$$

The stress-strain is related as-

(6) $$\left\{\bar{\sigma_{ij}}\right\}=\left\{C_{ij}\right\}\left\{\bar{\varepsilon_{i}}\right\}$$

Where, i, j varies from 1 to 6.

The Stiffness matrix ($C_{ij}$) obtained as,

(7) $$C_{ij}=\frac{\bar{\sigma_{ij}}}{\bar{\varepsilon_{ij}}}$$

After obtaining Stiffness matrix ($C_{ij}$), the Young's modulus is computed as-

(8) $$E_{1}=C_{11}-\frac{2C_{12}^{2}}{\left(C_{22}+C_{23}\right)}$$

(9) $$E_{2}=\frac{\left[2C_{11}\left(C_{22}+C_{23}\right)-2{C_{12}}^{2}\right]\left(C_{22}-C_{23}\right)}{\left(C_{11}C_{22}-{C_{12}}^{2}\right)}$$

The description of all the symbols mentioned to Sharma et al. [28].

**Fig. 4:**
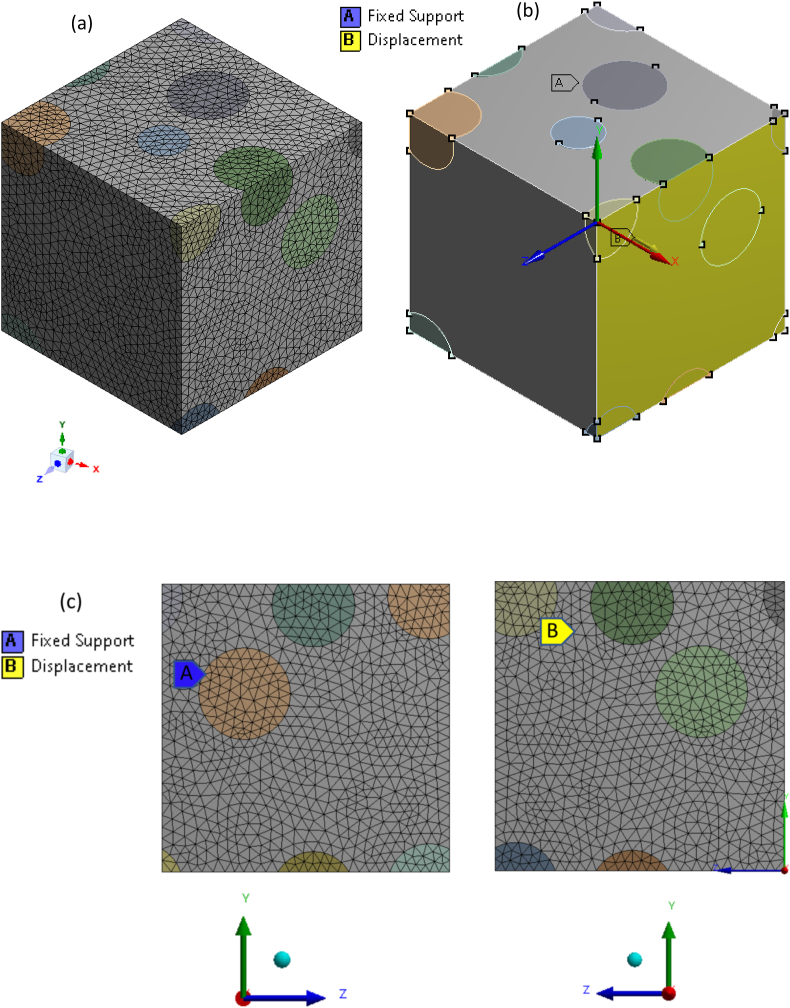
a) 3D Mesh view model; b) 3D view; c) 2D view of Boundary condition for one of the sample rHDPE/10% SiC.

**Fig. 5:**
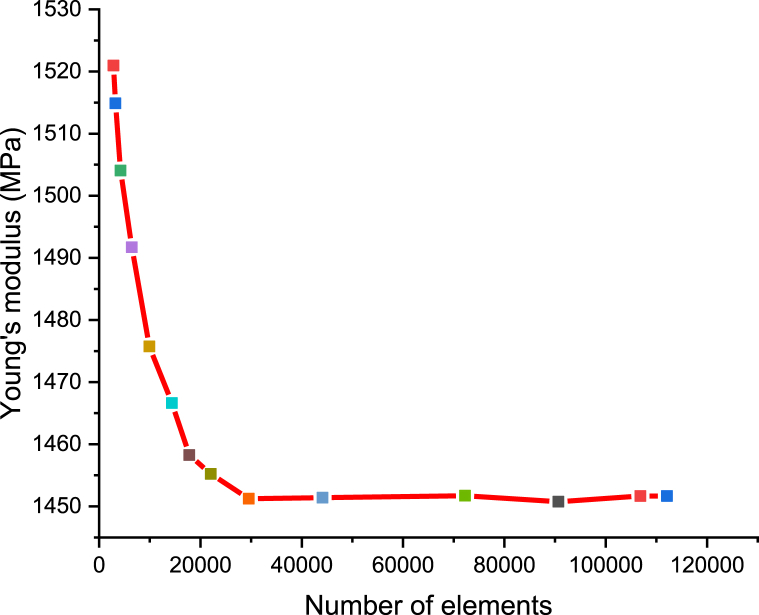
Mesh convergence of RVE for one of the sample rHDPE/10% SiC.

In RVE, the microstructure comprises of rHDPE matrix filled with randomly distributed SiC particles (spherical shape) of 50 μm size. A perfectly bonded topology is assumed between the matrix and fillers. The material property adopted for FEM analysis is described in section 2.1. The tetrahedral mesh elements and periodic boundary conditions were used for the composite as shown in Fig. 4a. The displacement back-side (i.e., negative x direction) face of the cuboid RVE was constrained. The displacement of 0.001 mm was allowed only along the front face (i.e., positive x-direction), as shown in Fig. 4b–c. To examine how the number of mesh components influences outcomes, a mesh convergence examination was carried out. Mesh convergence study for a 10 vol percentage rHDPE/SiC composite is shown in Fig. 5. The findings show that the results are close after 42450 element numbers, and it is seen that Young's modulus value decreases as the number of elements increases is in line with the literature [29]. Hence, close to 42450 mesh element numbers are used to analyse results for all the composites.

## 3 Results and discussion

**Fig. 6:**
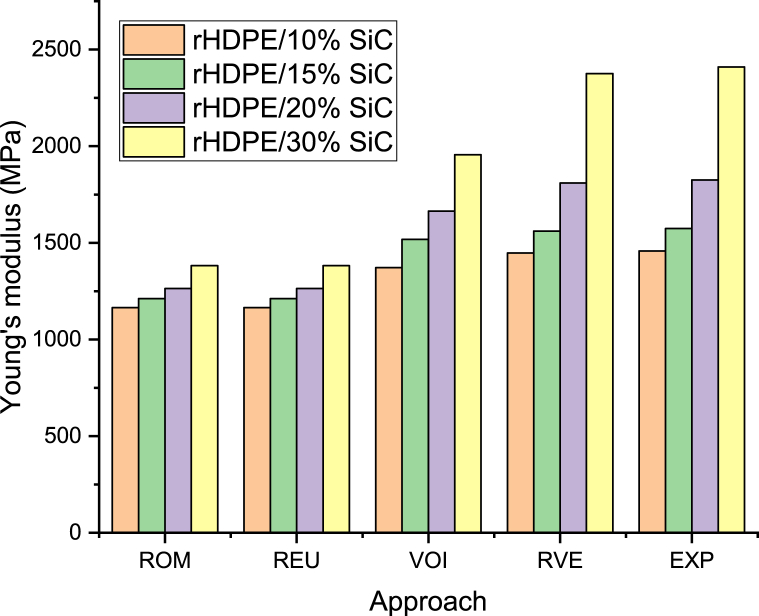
Young's modulus value through various approaches.

The representative volume element (RVE) is used to access the tensile modulus of recycled HDPE (rHDPE) reinforced with 10, 15, 20, and 30 vol % spherical shaped silicon carbide (SiC). Fig. 6 shows Young's modulus of rHDPE/10% SiC composite obtained through experimental tests along with the RVE technique and the three micro-mechanical models such as Rule of mixture (ROM), Reuss (REU), and Voight (VOI) model. The Young's modulus of rHDPE/10% SiC composite measured via experimental procedure is 1458 MPa, while the predicted values through ROM, REU, VOI, and RVE are 1165 MPa, 1165 MPa, 1372 MPa, and 1447 MPa respectively. Similarly, for the rHDPE/30% SiC composite, the evaluated Young's modulus value is 2410 MPa and the predicted values through ROM, REU, VOI, and RVE are 1382, 1382, 1956, and 2376 MPa. According to the abovementioned findings, the RVE closely and reliably predicted the value of Young's modulus compared to other theoretical techniques.

**Fig. 7:**
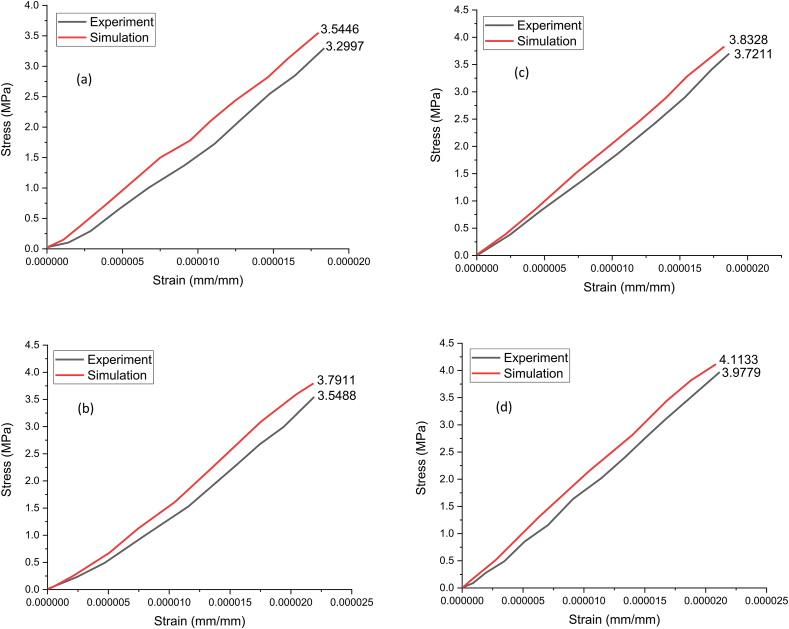
Experimental vs. simulation Stress-strain results of a) rHDPE/10% SiC; b) rHDPE/15% SiC; c) rHDPE/20% SiC; d) rHDPE/30% SiC.

**Fig. 8:**
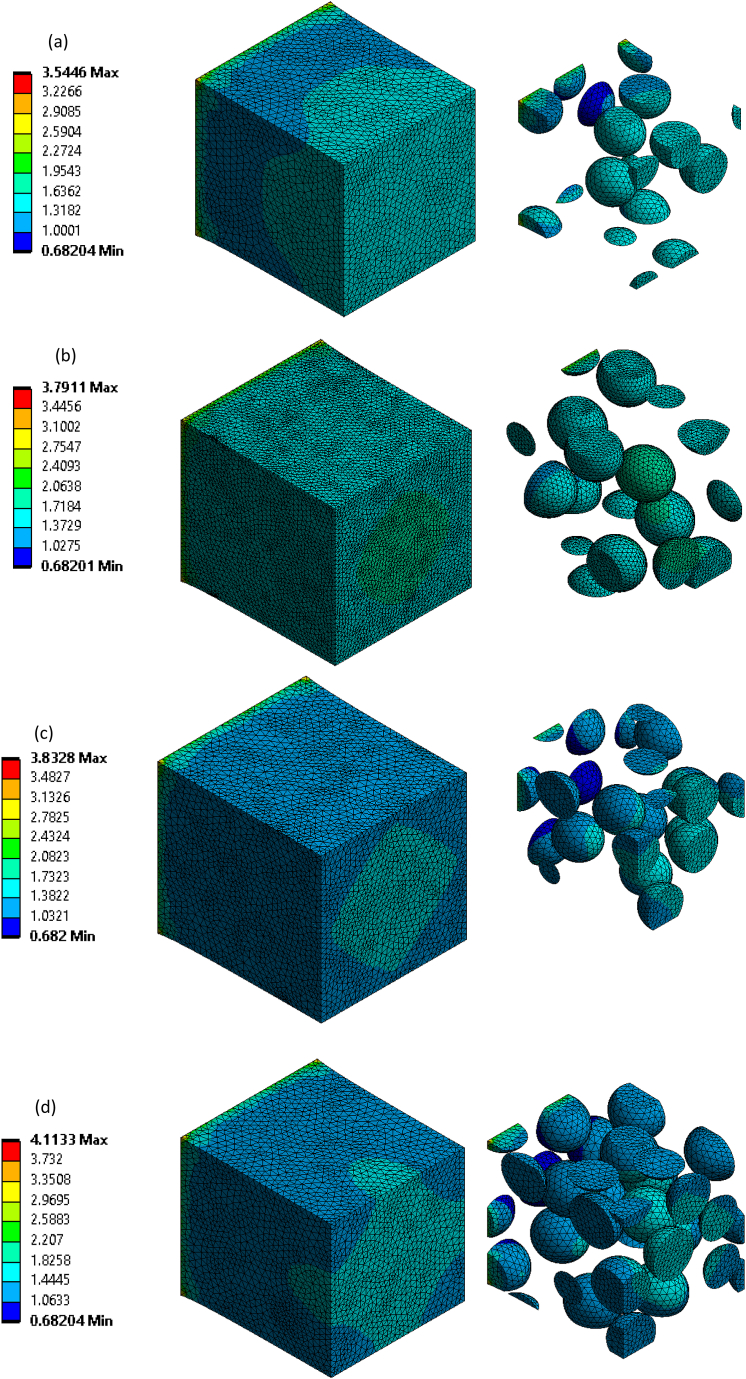
Simulation stress contour results of a) rHDPE/10%SiC; b) rHDPE/15%SiC; c) rHDPE/20%SiC; d) rHDPE/30% SiC.

Fig. 7a–d shows the stress vs. strain curve obtained through static structural simulation of imported RVE for all the composites. The maximum equivalent stress for rHDPE/10% SiC composite is 3.5446 MPa, which is increased by 7, 8, and 16% for rHDPE/15% SiC, rHDPE/20% SiC, and rHDPE/30% SiC, composite respectively. The reason for this is the structure of Silicon carbide (SiC), which is built with tetrahedral of carbon atoms at the core and shared with silicon atoms along its periphery as rigid bonding in the crystal lattice, which helps to achieve superior modulus value [30]. In addition, the further increase in volume % of SiC consequently improvises Young's modulus value. To validate the stress-strain results of RVE simulation, the results are compared with the experimental test value. It is observed that the error % are 7.1, 6.8, 3.1, and 3.4% for rHDPE/10% SiC, rHDPE/15% SiC, rHDPE/20% SiC and rHDPE/30% SiC composite respectively, which indicates the RVE simulation results are closely in line with the experimental values [31]. Fig. 8a–d shows stress contour results of 10, 15, 20, and 30% SiC-reinforced rHDPE composite. From Fig. 8a–d, it is clear that the matrix-filler system is uniformly stressed throughout and reaches its peak at the free end. It is also noted that the composites were stiffer and had increased stress value with increased SiC concentration.

## 4 Conclusion

The current study aims to use a FEM-based representative volume element (RVE) method to measure the tensile modulus of recycled high-density polyethylene (rHDPE) filled with 10, 15, 20, and 30 vol % randomly dispersed spherical-shaped silicon carbide (SiC). The tensile modulus determined by the RVE closely matches the findings of the experimental results. It is also noted a higher volume fraction of SiC has the excellent value of maximal equivalent stress of the composite. The error % observed between the simulation and the experimental values is less than 10%. The RVE method is used in the current study to take into account the randomly dispersed spherical-shaped silicon carbide (SiC) particles in the recycled high-density polyethylene (rHDPE) matrix. However, the impact of the locations and size of silicon carbide (SiC) particles is outside the purview of the current work, which will be carried out in the future investigation.

## Author contribution statement

Santosh Kumar Sahu: Conceived and designed the experiments; Performed the experiments; Analyzed and interpreted the data; Contributed reagents, materials, analysis tools or data; Wrote the paper. P. S. Rama Sreekanth: Conceived and designed the experiments; Analyzed and interpreted the data; Contributed reagents, materials, analysis tools or data.

## Funding statement

This research did not receive any specific grant from funding agencies in the public, commercial, or not-for-profit sectors.

## Data availability statement

The authors are unable or have chosen not to specify which data has been used.

## Declaration of competing interest

The authors declare no competing interests.
